# Predictors of discrepancies between fathers and mothers in rating behaviors of preschool children with and without ADHD

**DOI:** 10.1007/s00787-016-0897-3

**Published:** 2016-08-30

**Authors:** Lianne van der Veen-Mulders, Maaike H. Nauta, Marieke E. Timmerman, Barbara J. van den Hoofdakker, Pieter J. Hoekstra

**Affiliations:** 1 University of Groningen, University Medical Center Groningen, Department of Child and Adolescent Psychiatry, 660, 9700 AR Groningen, The Netherlands; 2 University of Groningen, Department of Clinical Psychology, Groningen, The Netherlands

**Keywords:** Behavior problems, Parental ratings, Discrepancy, Preschoolers

## Abstract

To examine child factors and parental characteristics as predictors of discrepancies between parents’ ratings of externalizing and internalizing behavior problems in a sample of preschool children with ADHD and behavior problems and in a nonclinical sample. We investigated correspondence and discrepancies between parents’ ratings on the externalizing and internalizing behavior problems broadband scales of the Child Behavior Checklist version for preschool children (CBCL/1.5–5). Parents of 152 preschool children, with ADHD and behavior problems (*n* = 72) and nonclinical children (*n* = 80), aged between 28 and 72 months (*M* = 47.26, SD = 12.7), completed the CBCL/1.5–5. Candidate predictors of discrepancy included the child’s age and sex, and parents’ levels of parenting stress, depressive mood, attention-deficit and disruptive behavior. Hierarchical multiple regression analyses were conducted. Correspondence between parents, both for ratings on internalizing and externalizing behavior problems, was high (*r* = .63–.77*)*. In the clinical sample, mothers rated the severity of externalizing behavior problems significantly higher than did fathers (*p* = < .001). Discrepancy between fathers and mothers on externalizing behavior problems was not predicted by child factors or interparental differences in psychopathology, but it was predicted by interparental differences in parenting stress (*R* ^2^ = .25, *p* < .001). This effect was significantly larger in the nonclinical sample (Δ*R* ^2^ = .06, *p* < .001). When parents disagree on the severity level of preschool children’s externalizing behavior problems, the clinician should take into consideration that differences in parenting stress might be involved.

## Introduction

Parents are the most important informants on children’s behavior problems, especially for preschool children, who spend most of their time at home. Guidelines for establishing diagnoses of attention-deficit and disruptive behaviors in preschool children advise to collect information from different informants and settings [1, 2] including the home setting. However, a growing body of evidence indicates that mothers and fathers may differ on their perceived levels of behavior problems of their child [3–5]. A better understanding of informant discrepancies will help interpret them correctly for assessment and treatment evaluation in both clinical practice and research [6].

There are two ways to explore the agreement between parental ratings, that is, through correspondence and discrepancy. Correspondence indicates the cohesion between informants’ ratings and focuses on whether informants’ ratings are correlated [7]. A high correlation between scores on (sub)scales of two informants means that the patterns of scores on (sub)scales are alike in shape and dispersion, but does not indicate anything about differences in level [8]. Thus, ratings may be highly correlated but still refer to different levels of severity of problems [9]. Furthermore, correspondence does not convey which informant reports more problems and on which type of problems [9]. The second method, discrepancy, provides an indication about the difference in levels of severity of problems between two raters.

Correspondence between fathers and mothers is usually in the high-to-moderate range (*r* = .45–.70), according to meta-analyses on interparental agreement on internalizing and externalizing behavior problems in mostly middle childhood aged children and adolescents. Highest levels of correspondence have been found on externalizing behavior problems (*r* = .62–.66) and lowest on internalizing behavior problems (*r* = .46–.59) [9–11]. In preschool children, results have been similar: a study on parental ratings of internalizing behavior problems among children, aged 3–8 years, referred for disruptive behavior problems, showed a moderate level of correspondence (*r* = .44) [12], while correspondence between parents on externalizing behavior problems in nonreferred preschool children was moderate to high (*r* = . 47–.74) [5, 13, 14].

Regarding discrepancy, a meta-analysis indicated that mothers tend to report more behavior problems in school-aged children than do fathers [9]. In addition, in two studies in preschool children, they were the mothers who reported more problems than did the fathers, with regard to ratings of internalizing behavior problems [12] and ratings of hyperactive and aggressive behavior [13]. In contrast to these findings, a comparison of parental ratings of preschool child behavior on the Strengths and Difficulties Questionnaire (SDQ) showed significantly higher hyperactivity, but not conduct behavior, ratings by fathers [5].

High correspondence between parents may be due to the fact that they both observe their child’s behavior in the same context, i.e., at home and from the same perspective of being the parent. Greater convergence between parental reports may signal children’s behavior being relatively independent of parental behavior and perspective. Furthermore, high correspondence may reflect consistency in the display of concerns about behavior problems across interactions with both parents at home [14]. However, discrepancies are common, and certainly also occur when informant (i.e., mother and father) reports are the result of rating scales with the same psychometric qualities. It could be hypothesized that more divergence between parents may be due to actual different behavior of the child in interaction with both parents, or to characteristics of the informants influencing the perception of, and concerns about child behavior. This last conceptual model on discrepancies [15] is the focus of this study, exploring child and parental characteristics as possible predictors for parental discrepancy.

Studies on this subject have mostly focused on internalizing behavior problems in parents, such as depressive mood and parental stress, but parental externalizing behavior problems have scarcely been investigated as a predictive factor. Concerning the role of parents’ internalizing problems in explaining the discrepancy on externalizing behavior problems in their children, one study concluded that parenting stress but not depression was linked with discrepancies in 7-to-9-year-old children with attention-deficit/hyperactivity disorder (ADHD) [3]. Parenting stress appeared to impact fathers differently than mothers when rating child ADHD symptomatology: fathers with lower levels of parenting stress tended to rate children’s ADHD and externalizing symptoms lower than did mothers with lower levels of parenting stress, whereas fathers with higher levels of parenting stress rated their child’s ADHD more severely compared with mothers with high levels of stress. Paternal depression was found to be a predictor of discrepancies between parents in nonreferred 3-year-old children (*n* = 196) with hyperactive behavior and attention problems [13]. Stress in fathers and alcohol misuse were found to be associated with higher paternal reports of externalizing behavior in nonreferred 4-to-6-year-old children than mothers [5]. Maternal factors were not investigated in that study.

Parents’ internalizing problems have also been investigated to explain parental discrepancies on internalizing behavior problems. A recent study [12] found that higher father stress ratings were associated with higher mother–father disagreement on co-occurring internalizing behavior problems, in children aged 3–8 (*n* = 181) with oppositional defiant disorder (ODD) and/or conduct disorder (CD). Depression in parents was not found to be predictive for discrepancy in that study. This contrasts to findings from a study on parental depression, in which fathers who were more depressed rated more internalizing problems in their first-grade school-age children, relative to mothers [16]. In a study on adolescents with internalizing behavior problems [17], mothers with higher levels of stress and depression rated higher levels of internalizing symptoms in their sons, compared to fathers, and fathers with higher levels of stress and anxiety rated higher levels of internalizing symptoms in their daughters, compared to mothers. In conclusion, levels of parental-rated parenting stress and depression, and sex of the child seem to be important factors regarding discrepancies between parents.

Externalizing behavior problems in parents have scarcely been investigated as a predictive factor for informants’ discrepancy on youth’s behavior, and as far as we know never in preschoolers. About 25–50 % of the children with ADHD have a parent with the same diagnosis [18]. On the one hand, parents with ADHD symptoms may be more accepting of ADHD characteristics in young children [19]. On the other hand, it may also be the case that parents with ADHD symptoms will more easily become distressed and overwhelmed by the hyperactive or oppositional behavior of their child. One study on the influence of maternal ADHD on reports of ADHD symptoms in school-age children and adolescents found no evidence for such influence [20]. Paternal antisocial behavior and maternal substance abuse were also found to be unrelated to higher discrepancy with other informant’s ratings of behavior in adolescents [8]. However, parental anger predicted variance in ratings in externalizing and internalizing behavior problems in first-grade school-age children. More angry parents reported more externalizing and internalizing behavior problems [16].

Child characteristics may also play a role in explaining discrepancies between parents. With regard to the child’s sex, it is noteworthy that the discrepancy in ratings of young children’s internalizing symptoms between fathers and mothers was found to be larger for girls than for boys [12], with mothers reporting most symptoms for girls. In a study on parental reports of preschool children’s behavior, fathers were four times more likely to report hyperactive behavior among boys compared with girls [5]. A recent meta-analysis [11] found no age-effect on levels of correspondence between raters for both externalizing and internalizing behavior problems in younger (i.e., 10 years and younger) and older (i.e., 11 years and older) children. Correspondence between parents’ ratings of externalizing behavior problems in 12-month-old children was found to be lower than in 24-to-36-month-old children [21].

The aim of this study was to explore different factors in children and parents that may be related to variations in reporting different levels of severity of externalizing and internalizing behavior problems. In this study, we examined the correspondence and discrepancy between parents on internalizing and externalizing behavior problems in two samples, namely, a clinical sample of preschool children with ADHD and disruptive behavior problems who had been referred to an outpatient mental health clinic, and in a nonclinical sample of preschool children. Consistent with previous research, we expected moderate to large levels of correspondence between raters. Regarding discrepancy, we expected mothers’ ratings of behavior problems in children to be higher than fathers’ ratings. We hypothesized that parental stress would be of influence on parental ratings of children’s behavior problems, with parents experiencing higher levels of stress reporting higher levels of problematic child behavior. We did not have hypotheses on the predictive value of child factors and parental characteristics on discrepancy between parents, since previous findings on these subjects were not consistent. Moreover, we expected parental reports on children’s behavior problems to be particularly discrepant in case parents rated themselves more deviant from the other parent. Therefore, we not only focused on characteristics of fathers and mother as such, but also on the role of the difference in characteristics between parents.

## Method

### Participants and procedure

The total sample (*N* = 152) consisted of a clinical (*n* = 72) and a nonclinical sample (*n* = 80). Most of the children were Caucasian (89 %). Children in the clinical sample had been referred to an outpatient mental health clinic, because of disruptive behavior problems. All these children had received an ADHD diagnosis, based on a semi-structured interview with the parents (i.e., the Dutch version, adapted for young children, of the Parent Interview for Child Symptoms PICS-4; [22]) and the teacher/caregiver (i.e., the Dutch version of the Teacher Telephone Interview: TTI; [23]) by an experienced clinician. Assessment of behavior problems was based on the same semi-structured interviews and on mother’ reports of child behavior on the Eyberg Child and Behavior Inventory (ECBI [24]). Children in the clinical sample participated in a study into the treatment of ADHD symptoms and behavior problems in preschool children. This study was ethically approved and parents gave informed consent.

The clinical sample consisted of 58 boys (81 %) and 14 girls (19 %) aged between 32 and 71 months (*n* = 54.84, SD = 11.0) at the date of rating, who all had two caretakers. All primary caretakers were mothers, including one foster mother and 71 biological mothers. Secondary caretakers of 64 children (89 %) were biological fathers, of six children (8 %) stepfathers, of one child a foster father (1.5 %), and of one child a grandmother (1.5 %). Eight mothers (11 %) were single mothers, with the father living elsewhere (who still rated the child in this study).

The nonclinical sample included 80 control children with two caretakers, recruited from schools and childcare centers in the northern region of the Netherlands, 42 boys (53 %) and 38 girls (47 %) aged between 28 and 72 months (*M* = 47.26, SD = 12.7). All primary caretakers were mothers including one stepmother. Secondary caretakers of 77 children (95 %) were biological fathers, two stepfathers (3 %), and one second mother (2 %). Six mothers (7.5 %) were single mothers with the father living elsewhere (who still rated the child in this study).

All parents in the nonclinical sample filled in the measurements at home. Parents in the clinical sample filled in the rating scales at home or at the clinic, just before start of the treatment. All parents were instructed to fill in the questionnaires separately and independently.

## Measures

### Child Behavior Checklist (CBCL), preschool version

Both caretakers on the preschool CBCL rated children’s behavior problems for children aged 1½–5. The preschool CBCL 1½–5 has two empirically based broadband subscales: externalizing (24 items) and internalizing (36 items) behavior problems [25]. Each item can be scored with 0 (not true), 1 (sometimes true), or 2 (very true or often true). The Externalizing Problems scale (CBCL-EXT) consists of two scales: attention problems and aggressive behavior. The Internalizing Problems scale (CBCL-INT) consists of four scales: emotionally reactive, anxious/depressed, somatic complaints, and withdrawn. Reliability and validity of the CBCL are well-documented [26]. Cronbach’s coefficient α (which provides a lower bound for the reliability of the scale) equaled .95 for the externalizing subscale and .89 for the internalizing subscale in our total sample.

### Parenting Stress Index (PSI), short form

We measured presence of parenting stress using the Dutch, short version of the Parenting Stress Index (PSI) [27], the Nijmeegse Ouderlijke Stress Index short form (NOSIK) [28]. The NOSIK is a parent-report instrument and consists of 25 parenting stress related items with answers on a 6-point Likert scale ranging from 1 (totally disagree) to 6 (totally agree) resulting in a total stress score. The NOSIK contains items from two different domains: the parental (11 items) and child (14 items) domain, as well as a total scale. In line with previous research [29], five items from the child domain (“Mood” and “Distractibility/Hyperactivity”) were removed from the analyses because of possible confound with the problem behaviors in the CBCL. Reliability and criterion validity of the NOSI-K are high [28]. Cronbach’s coefficient α equaled .96 for our total sample on the total scale of the adapted PSI.

### Beck’s depression inventory (BDI)

We used the 21-item BDI [30]. A four-point Likert scale indicates the degree of severity for every item from 0 (not at all) to 3 (very true). Internal consistency is typically very high, and high concurrent validity ratings are given between the BDI and other depression instruments [31]. Cronbach’s coefficient α equaled .87 for our total sample.

### Adult ADHD Rating Scale (AARS)

The AARS [32] is an 18-item adult self-report measure of ADHD symptoms. Ratings are on a 4-point Likert scale ranging from 0 (never or rarely) to 3 (very often). In this study, we used the total score for mothers and fathers on the AARS. Validity of the scale was suggested by high correlations between subjects and informant ratings [33]. Cronbach’s coefficient α equaled .93 for our total sample.

### Subtypes of Antisocial Behavior Questionnaire (STAB)

We assessed parental antisocial behavior with the STAB [34]. The STAB contains 32 self-report questions on aggressive behavior during lifetime. Items are scored on a 5-point Likert scale ranging from 1 (never) to 5 (nearly all the time). The STAB has three subscales: physical aggression, social aggression, and rule-breaking behavior. In this study, we used the total score for each parent on the STAB. Norms are not yet available. There is initial evidence of the factorial validity, internal consistency, and criterion-related validity of the STAB scales [34]. Cronbach’s coefficient α was .89 for our total sample.

### Statistical analyses

In the case of more than half items missing, the scale was discarded from analyses. If there were guidelines available on how to deal with missing items, we have followed these guidelines. In the case of no such instructions and there were less than half missing values for a scale, these values were replaced with the mean of other items of the scale. Less than 1 % item scores were missing on the scales included in the analyses. We performed available case analyses.

Because of two distinct subsamples (a clinical sample and a nonclinical sample), we expected outliers. Therefore, we conducted all analyses on a data set not corrected for outliers, as well as on a data set in which we replaced discrepancies of more than two standard deviations by the mean plus or minus two standard deviations. Such discrepancies were reported by less than 4 % of the sample. Analysis results are reported of the uncorrected data. Significant differences in results with the corrected data were not found.

As raw scores reflect the actual distribution of symptom behavior on the CBCL-EXT and CBCL-INT, we used raw scores for statistical analysis [26]. We explored correspondence between parents on the CBCL-EXT and CBCL-INT with Pearson Product Moment Correlations. We assessed differences in mother–father correlation coefficients between CBCL-EXT and CBCL-INT and between the two subsamples using Fisher r-to-z transformations. We conducted paired *t* tests to assess parental discrepancy on both scales and to compare differences between parents on CBCL-EXT and CBCL-INT in both subsamples. We explored discrepancies between parents on parenting stress and parental psychopathology using independent *t* tests. *p* values were adjusted with the Holm–Bonferroni procedure, to correct for multiple testing. Corrected values are reported. The correction was applied per sample, for all measures per sample [35].

Two outcome difference scores were constructed: the subtraction of fathers’ raw scores from mother’s raw scores on the externalizing (D-CBCL-EXT) and internalizing (D-CBCL-INT) behavior scale of the CBCL. Furthermore, four predictor difference scores were constructed: the subtraction of fathers’ raw scores from mother’s raw scores on the BDI (D-BDI), PSI (D-PSI) AARS (D-AARS), and STAB (D-STAB).

Note that we used raw difference scores, in line with Laird [36], rather than difference scores based on standardized scores (i.e., *z*-scores). Raw difference scores can be applied here, as we have the same rating scale for mothers and fathers. The use of *z*-scores would be necessary when different measures would be available for mothers and fathers [37].

We performed correlation analyses to investigate the strength of the linear relationships between the two outcome difference scores (D-CBCL-EXT and DCBCL-INT) and all possible predictors (children’s age and gender, mean raw scores for fathers and mothers on the parental factors (BDI, PSI, AARS, and STAB) and their raw difference scores (D-BDI, D-PSI, D-AARS, and D-STAB).

To examine possible predictors for observed discrepancies between parents on CBCL-EXT and CBCL-INT, a series of multiple regression analyses were performed. The series was performed separately with D-CBCL-EXT and D-CBCL-INT as the dependent variable. In the first step, a regression analysis was conducted on the total sample, with the dummy variable ‘sample’ (0 = nonclinical sample, 1 = clinical sample), the child’s age and the dummy variable ‘sex’ (0 = boy, 1 = girl) entered as predictors. In the second step, two multiple regression analyses were conducted with parental factors added to significant child predictors as possible predictors for discrepancies. The first regression was conducted with separate mothers and fathers scores on the parental factors (PSI, BDI, AARS, and STAB) and the second one with the four difference scores on these rating scales. As Laird [36] has recommended, the multi-informant mean was included as a predictor for the regression analyses with difference scores as predictors. Finally, we added an interaction term with the dummy variable ‘sample’ to investigate if there was an extra effect of significant predictors in one of the subsamples. Predictors that were not significant were removed from the analyses.

## Results

### Correspondence and discrepancy between parents

In both subsamples, parents highly agreed on the ratings of their child’s externalizing and internalizing behavior problems. Parental agreement on the CBCL-EXT in the clinical sample (*r* = .77, *p* < .001) was significantly higher (*z* = 1.68*, p* = .046) than agreement in the nonclinical sample (*r* = .63, *p* < .001). Parental agreement on the CBCL-INT in the clinical sample (*r* = .74, *p* < .001) did not significantly differ from that in the nonclinical sample (*r* = .67, *p* < .001).

Summary measures of the mother and father child and self-ratings are listed in Table 1. Mothers in the clinical sample rated significantly higher on the CBCL-EXT than did fathers *t*(71) = 4.650, *p* < .001. Mothers and fathers did not differ on CBCL-INT in any of the samples.

**Table 1:** Mother and father child and self-ratings

Variable	Mothers				Fathers				*p*
	*n*	*M*	Range	SD	*n*	*M*	Range	SD	
*Clinical sample*									
Child’s externalizing behavior problems (CBCL-EXT)	72	30.77	36	7.26	72	27.89	37	8.14	.000***
Child’s internalizing behavior problems (CBCL-INT)	72	17.34	36	7.25	72	16.16	38	8.80	.333
Parental ADHD (AARS)	69	13.77	40	9.57	68	12.15	43	9.91	.664
Parental depression (BDI)	70	9.03	27	5.72	68	4.92	24	5.32	.000***
Parental antisocial behavior (STAB)	70	39.75	24	5.83	67	40.41	36	8.14	.664
Parenting stress (PSI)	70	67.74	75	17.80	68	59.26	84	19.67	.036*
*Nonclinical sample*									
Child’s externalizing behavior problems (CBCL-EXT)	80	10.17	31	7.34	80	10.27	31	7.52	1.0
Child’s internalizing behavior problems (CBCL-INT)	78	6.67	22	5.05	78	7.43	27	6.41	.99
Parental ADHD (AARS)	72	8.52	38	7.40	71	7.74	41	6.93	1.0
Parental depression (BDI)	72	5.38	18	4.31	70	4.14	32	6.11	.99
Parental antisocial behavior (STAB)	70	37.57	21	4.87	71	39.14	41	6.83	.99
Parenting stress (PSI)	79	31.86	49	11.58	79	30.98	50	12.33	1.0

*CBCL-INT* Child Behavior Checklist, Internalizing scale, *CBCL-EXT* Child Behavior Checklist, Externalizing scale, *BDI* Beck’s depression inventory, *AARS* Adult ADHD Rating Scale, *STAB* Subtypes of Antisocial Behavior Questionnaire, *PSI* Parenting Stress Index

*t* test values, Holm–Bonferroni adjusted *p* values * *p* < .05, ** *p* < .01, *** *p* < .001

In the clinical sample, mothers rated themselves as more depressive and experiencing more parental stress than did fathers. Mothers in the clinical sample rated significantly more depressive mood *t*(140) = 4.299, *p* < .001, parenting stress *t*(116) = 14.368, *p* < .001, ADHD behaviors *t*(139) = 3.577, *p* < .001, and antisocial behaviors *t*(138) = 2.357, *p* = .020, than mothers in the nonclinical sample. Fathers in the clinical sample rated significantly more parenting stress *t*(109) = 10.252, *p* < .001 and ADHD *t*(137) = 2.904, *p* = .012 than fathers in the nonclinical sample, and there were no significant differences on depressive symptoms and antisocial behavior.

Results on correspondence between possible predictors and interparental rating discrepancies on children’s externalizing and internalizing ratings are reported in Table 2. In the clinical sample, only the difference between parents on self-rated parenting stress was significantly associated with discrepancy on ratings of CBCL-EXT. In the nonclinical sample, all difference score predictors, except D-BDI and D-CBCL-EXT, were significantly associated with discrepancy between parent ratings of both internalizing and externalizing behavior problems in children. Age and sex of the child were not associated with discrepancies on CBCL-EXT and CBCL-INT.

**Table 2:** Correlations between possible predictors and interparental rating discrepancies on children’s externalizing (Child Behavior Checklist, externalizing scale; CBCL-EXT) and internalizing ratings (Child Behavior Checklist, Internalizing scale; CBCL-INT)

Predictors	Difference score externalizing behavior problems (D-CBCL-EXT)		Difference score internalizing behavior problems (D-CBCL-INT)	
	*n*	*r*	*n*	*r*
*Clinical sample*				
Mothers’ ADHD (AARS)	69	.05	69	−.06
Fathers’ ADHD (AARS)	68	−.37*	68	−.33*
Difference score mother–father ADHD (D-AARS)	67	.26	67	.17
Mothers’ depression (BDI)	70	−.07	70	.09
Fathers’ depression (BDI)	68	−.30	68	−.14
Difference score mother–father depression (D-BDI)	67	.19	67	.18
Mothers’ antisocial behavior (STAB)	70	.05	70	.09
Fathers’ antisocial behavior (STAB)	67	−.08	67	−.20
Difference score mother–father antisocial behavior (D-STAB)	66	.09	66	.24
Mothers’ parenting stress (PSI)	70	.10	70	.19
Fathers’ parenting stress (PSI)	68	−.36*	68	−.08
Difference score mother–father parenting stress (D-PSI)	67	.43***	67	.25
Child’s sex	72	.15	72	.17
Child’s age	72	−.06	72	−.01
*Nonclinical sample*				
Mothers’ ADHD (AARS)	70	.16	70	.16
Fathers’ ADHD (AARS)	70	−.28	70	−.56***
Difference score mother–father (D-AARS)	71	.38*	70	.56***
Mothers’ depression (BDI)	72	.09	70	.03
Fathers’ depression (BDI)	70	−.20	69	−.41***
Difference score mother–father (D-BDI)	70	.31	69	.52***
Mothers’ antisocial behavior (STAB)	70	−.01	70	−.09
Fathers’ antisocial behavior (STAB)	70	−.44***	67	−.52***
Difference score mother–father (D-STAB)	69	.41*	68	.43*
Mothers’ parenting stress (PSI)	79	.14	70	.13
Fathers’ parenting stress (PSI)	79	−.44***	68	−.34*
Difference score mother–father (D-PSI)	78	.56***	76	.49***
Child’s sex	80	.10	80	.20
Child’s age	80	−.07	80	−.17

*BDI* Beck’s Depression Inventory, *AARS* Adult ADHD Rating Scale, *STAB* Subtypes of Antisocial Behavior Questionnaire, *PSI* Parenting Stress Index, *M* mothers score, *F* fathers score, *D* difference score

Pearson correlations, Holm–Bonferroni adjusted *p* values * *p* < .05, ** *p* < .01, *** *p* < .001

Looking at mothers’ and fathers’ scores on the parental factors, we only found significant associations between fathers’ scores and discrepancies between parents’ ratings of behavior problems in children. In the clinical sample, we found a significant association between parenting stress in fathers, and discrepancy on externalizing behavior problems. Furthermore, we found an association between paternal ADHD and discrepancy between parents on both externalizing and internalizing behavior. In the nonclinical sample, we found significant associations between all fathers’ scores on parental factors and differences between parents on their ratings of children’s internalizing behavior problems. We also found associations between fathers’ scores on parenting stress and antisocial behavior, and disagreement between parents on externalizing behavior problems.

### Predictors of discrepancy

First, sample (0 = nonclinical sample, 1 = clinical sample), the child’s age, and sex (0 = boy, 1 = girl) were entered in the regression analysis to investigate a possible relationship between these three child factors and rating discrepancies. Results are reported in Tables 3 and 4. In Step 1, the following appeared: D-CBCL-EXT, differences between parents with regard to externalizing problems, was predicted by sample but not by age or sex. D-CBCL-INT, differences between parents on internalizing behavior problems, was predicted by sample and sex, but not by age.

**Table 3:** Hierarchical multiple regression analyses predicting parental rating discrepancies on externalizing (Child Behavior Checklist, externalizing scale; CBCL-EXT) and internalizing behavior problems (Child Behavior Checklist, externalizing scale; CBCL-EXT) with child factors and parental factors

Predictor	Discrepancies on externalizing behavior problems (D-CBCL-EXT)			Discrepancies on internalizing behavior problems (D-CBCL-INT)		
	*R* ^2^	SE *B*	*β*	*R* ^2^	SE *B*	*β*
Step 1	.06***			.06***		
Constant		.66			.77	
Sample: 0 = nonclinical sample, 1 = clinical sample		.96	.25**		.94	.22**
Child’s Sex: 0 = boy, 1 = girl					.99	.18*
Step 2	.25***			.32***		
Constant		1.22			2.28	
Sample: 0 = nonclinical sample, 1 = clinical sample		1.43	.28**			
Child’s Sex: 0 = boy, 1 = girl					.88	.20**
Mothers’ parenting stress (PSI)		.03	.50***		.19	.40***
Fathers’ parenting stress (PSI)		.03	−.63***			
Fathers’ ADHD (AARS)					.05	−.40***
Fathers’ antisocial behavior (STAB-F)					.06	−.20*

*AARS* Adult ADHD Rating Scale, *STAB* Subtypes of Antisocial Behavior Questionnaire, *PSI* Parenting Stress Index

* *p* < .05, ** *p* < .0, *** *p* < .001

**Table 4:** Hierarchical multiple regression analyses predicting parental rating discrepancies on externalizing (Child Behavior Checklist, externalizing scale; CBCL-EXT) and internalizing behavior problems (Child Behavior Checklist, externalizing scale; CBCL-EXT) with child factors and difference scores on parental factors

Predictor	Discrepancies on externalizing behavior problems (D-CBCL-EXT)			Discrepancies on internalizing behavior problems (D-CBCL-INT)		
	*R* ^2^	SE *B*	*β*	*R* ^2^	SE *B*	*β*
Step 1	.06***			.06***		
Constant		.66			.77	
Sample: 0 = nonclinical sample, 1 = clinical sample		.96	.25**		.94	.22**
Child’s Sex: 0 = boy, 1 = girl					.99	.18*
Step 2	.27***			.31***		
Constant		.60			.88	
Sample: 0 = nonclinical sample, 1 = clinical sample		.90	.13		.97	.28**
Child’s Sex: 0 = boy, 1 = girl					.93	.21**
Differences in parenting stress (D-PSI)		.03	.47***		.03	.18*
Differences in parental ADHD (D-AARS)					.04	.18*
Multi-informant mean parental ADHD (AARS)					.07	−.28***
Differences in antisocial behavior (D-STAB)					.05	.19*
Step 3	.33***			.34***		
Constant		.58			.86	
Sample: 0 = nonclinical sample, 1 = clinical sample		.88	.19*		.96	.29**
Interaction sample and D-PSI		.06	−.48***			
Differences in parenting stress (D-PSI)		.05	.87***		.03	.17*
Interaction sample and D-AARS					.08	−.29*
Differences in parental ADHD (D-AARS)					.06	.43**
Multi-informant mean parental ADHD (AARS)					.07	−.29***
Child’s Sex: 0 = boy, 1 = girl					.92	.20*
Differences in antisocial behavior (D-STAB)					.05	.15^1^

*AARS* Adult ADHD Rating Scale, *STAB* Subtypes of Antisocial Behavior Questionnaire, *PSI* Parenting Stress Index

* *p* < .05, ** *p* < .0, *** *p* < .001.^1^ = trend significant *p* < .1

In Step 2, separate mothers’ and fathers’ scores on parental factors were added to relevant child factors as possible predictors for D-CBCL-EXT and D-CBCL-INT (see Table 3). For D-CBCL-EXT, it appeared that parents disagreed more on ratings of their child’s externalizing behavior problems with increasing parenting stress in mothers, and decreasing parenting stress in fathers. Due to non-significance, ADHD, depressive mood, and antisocial behavior in parents were discarded from further analyses. There was no interaction effect for the separate samples (nonclinical and clinical, i.e., ADHD sample) and mothers’ scores on parenting stress, and no interaction effect for sample and fathers’ scores.

For D-CBCL-INT, it appeared that parents disagreed more on ratings of internalizing behavior problems with lower levels of paternal ADHD and paternal antisocial behavior, and with high levels of parenting stress in mothers. Sex remained a significant predictor, indicating greater discrepancy on girls. There were no differences regarding this pattern between the two subsamples. Sample, parenting stress in fathers, parental depressive mood, ADHD in mothers, and antisocial behavior in mothers were rejected from further analysis.

Finally, multiple regression analyses were conducted with difference scores on parental factors added to significant child factors as possible predictors. Results are reported in Table 4. For D-CBCL-EXT, it appeared that parents disagreed more on ratings of their child’s externalizing behavior with increasing differences in parenting stress, accounting for 25 % of the variance. That is, higher difference scores on parenting stress were associated with a higher positive score on D-CBCL-EXT. As indicated by the interaction between sample and D-PSI in Step 3 (see Table 4), this effect was stronger in the nonclinical sample than in the clinical sample. Differences in psychopathology between parents were not predictive of discrepancies on ratings of externalizing behavior problems of the children and therefore discarded from analyses.

For D-CBCL-INT, it appeared that parents disagreed more on the level of the internalizing behavior problems of their child with increasing differences in parenting stress, increasing differences in parental-rated ADHD, decreasing multi-informant mean of parental-rated ADHD, and increasing differences in antisocial behavior. In total, this predicted 22 % of variance in discrepancies. That is, a higher multi-informant mean on the AARS was associated with less difference or a more negative difference score on internalizing behavior problems. A higher difference score on parental ADHD, parenting stress, and antisocial behaviors, meaning mothers rating themselves as having more problems than fathers, was associated with a higher D-CBCL-INT, meaning mothers rating more internalizing behavior problems than fathers. A higher negative difference score on these parental factors, meaning fathers rating themselves as having more problems than mothers, was associated with a more negative D-CBCL-INT, meaning fathers assessing more internalizing behavior problems in their child. Differences in depressive mood between parents were not predictive for discrepancies on ratings of internalizing behavior problems in children. As indicated by the interaction between sample and D-AARS in Step (see Table 4), there was a stronger effect of differences in parent rated ADHD in the nonclinical sample, but not for differences in antisocial behavior or parenting stress.

## Discussion

In this study, we examined the correspondence and discrepancy between parents on internalizing and externalizing behavior problems in a clinical and a nonclinical sample of preschool children. Our main finding regarding correspondence was that correspondence between parents on ratings of both internalizing and externalizing behavior problems in their children was high, which is in line with previous findings [9–11, 13] and our hypothesis. The observed correlations of .67–.74 between mother’s and fathers’ ratings of children’s internalizing behavior problems and of .63–.77 between parents’ ratings of externalizing problems were higher than correlations that have been found in previous studies. Although we instructed parents to fill in the assessments separately, it cannot be entirely excluded that some of them failed to do so.

With respect to discrepancy, it appeared that mothers in the clinical sample rated significantly higher levels of externalizing behavior problems than fathers, which is in line with our hypothesis. This effect was not found in the nonclinical sample. This parental discrepancy seems to be in contrast with findings of a meta-analysis, where no significant discrepancies between parents have been found [9]. However, the meta-analysis only included four studies with preschool children. Given that mostly mothers are primary caretakers and preschool children spend most of their time at home, it may be that particularly, mothers of young children, referred for disruptive behavior problems, may observe more problematic behavior than do fathers. Furthermore, disruptive behavior is common in preschool children and young children with ADHD exhibit even more noncompliant and inappropriate behavior than children without ADHD [38]. Perhaps, mothers struggle more to cope with the demands of parenting noncompliant children and this may influence mothers’ ratings of behavior problems in children different from fathers’ ratings, who generally spend less time with their children.

In contrast to the study of Mascendaro [12], we found no significant parental discrepancy on levels of internalizing behavior problems across both samples. The finding in that study was based on a clinical sample (*n* = 129) of children aged 3–8. Perhaps our finding regarding the absence of parental discrepancy on levels of internalizing behavior problems was due to a smaller clinical sample size (*n* = 72) and a lower age (2.5–6 years) of the participants. Internalizing behavior problems are difficult to observe in younger children, especially when they also have externalizing behavior problems.

Parental parenting stress was associated with discrepancy in ratings of externalizing behavior problems in all samples: high maternal parenting stress was related to increased differences between parental ratings of externalizing behavior problems, while high paternal parenting stress decreased the differences. If parents were more alike in parenting stress levels, they agreed more on ratings of their children’s externalizing behavior problems.

In case fathers experienced higher levels of parenting stress than mothers, they also reported more externalizing behavior problems. Langberg [3] investigated parent agreement on ratings of ADHD in children, aged between 7 and 9 years, and found that fathers with low parental stress rated their children’s ADHD and externalizing problems lower than mothers. However, as stress in fathers increased, they rated their children’s behaviors more severely than mothers. In our study, mothers with elevated stress rated more externalizing behavior problems than fathers, even when they both reported high parenting stress levels. This discrepancy with the results in Langberg’s study may be due to the different ages of participants in both studies and to the fact that mothers spend more time with their young children than fathers.

In general, parents in the nonclinical sample rated about generally low levels of externalizing behavior problems, with equal ratings between parents. Furthermore, the difference between parents in level of parenting stress predicted 25 % of variance in parental disagreement on ratings of children’s externalizing behavior problems, not only in the clinical sample, but also in the nonclinical sample. In the latter sample, the effect of differences in parenting stress on discrepancy in ratings of children’s externalizing behavior problems was significantly stronger than in the clinical sample. This may be due to many D-CBCL-EXT scores around zero in that sample and a higher variability in the clinical sample. Overall, the association between parenting stress and parental ratings on children’s externalizing behavior problems appears to be consistent, not only across caretakers but also across samples.

As mentioned before, the PSI contains items from two different domains: the parental and child domain. A recent meta-analysis on parenting stress in families of children with ADHD [39] showed that mothers only perceived to a small extent more parenting stress related to factors within the child, compared to fathers. In contrast with these findings, mothers in our clinical sample both reported significant more child and parent domain-related stress, than did fathers. Additional regression analyses [results available upon request] showed that in this sample, only differences in the adapted child domain of the PSI short form significantly contributed to the prediction of discrepancy on externalizing behavior problems. The adapted child domain consisted of five items of the subscale “Demandingness,” one item of “Adaptability,” three items of “Acceptability,” and one item of “Reinforcement.” Apparently, when parents experience their child’s behavior problems as demanding and difficult to accept, they tend to rate more externalizing behavior problems. This finding is in line with previous findings that showed the child domain of the PSI to be a better predictor of parent-reported behavior problems in children than the parent domain [40].

In addition, in line with previous findings [39, 41], we found ADHD in children to be associated with higher rates of parental psychopathology and parental stress; however, differences in parental psychopathology did not attribute to discrepancy in parental ratings of children’s externalizing behavior problems. This may have been due to a high correspondence between parental stress and parental psychopathology and/or our relatively small sample size. However, our findings are in line with previous findings demonstrating that the parent domain of the PSI, which measures emotional well-being of parents, did not predict parental ratings of child adjustment [40].

Discrepancy in parent reports of behavioral problems may reflect real-life differences in child behavior and parental perspectives of that behavior. Children may behave differently in various contexts [14], eliciting varying amounts of parenting stress. On the other hand, discrepancy between parents may reflect observations of the same child behaviors from different perspectives or moods. Parenting stress may draw parental attention to children’s oppositional behavior, resulting in elevated ratings of disruptive behavior [39]. Discrepancy between parents on ratings of children’s disruptive behaviors may also be related to the time spend with the child. In our study, all primary caretakers were mothers. Brown et al. [42] found that fathers generally spent less free time with temperamental children than mothers. Perhaps spending more time with a demanding child mediates more parenting stress, resulting in a less sensitive and responsive parent and more disruptive child behavior. In conclusion, differences in parenting stress and related discrepancy on ratings of children’s disruptive behaviors may be mediated by various factors. Further research on these mechanisms should be conducted.

## Limitations

First, our study was conducted with a relatively small sample size. This may explain why we did not find other significant predictors than parenting stress for discrepancy between parents. Second, children in the nonclinical sample were on average 7.58 months younger than in the clinical sample. This difference may have been of influence on correspondence and discrepancy of parental ratings. Finally, the clinical group included only 14 girls (i.e., 19 %), which precluded a strong test of gender effects.

This study did not include an external criterion variable for clinical levels of problem behavior, such as a clinical judgment based on a semi-structured interview. In the case of an external criterion variable, one could examine interactions of that outcome variable with convergence and divergence between raters [15], for instance, if divergence and convergence between mothers’ and fathers’ ratings on the CBCL are related to the judgment of the clinician on child’s behavior problems. Future research on informant discrepancies as predictors of external indicators of psychosocial functioning could provide additional important information on this subject.

## Clinical implications

We found that fathers and mothers in community samples rate their child’s behavior problems equally. In general, they do not differ in their ratings of externalizing and internalizing behavior problems. However, if parents perceive different levels of parental stress, discrepancy may occur on ratings of children’s externalizing behavior problems, both in a clinical and a nonclinical sample. The difference score on parental stress can be interpreted as “an estimate of the differential predictive ability of informants” [36]. When parents rate different levels of parental stress, the predictive ability of one parent for the other decreases on ratings of externalizing behavior problems in their children. For clinicians, it is necessary to consider possible discrepancy in parental stress in the assessment of preschool children’s behavior problems.

When making clinical decisions, clinicians tend to rely on mothers as the best informant, because they spend most of the time with the child. There is, however, no evidence for such a preference [43]. Our study shows that choosing for the mother as the only informant on rating scales may result in higher levels of severity on externalizing behavior problems, especially when mothers experience more parenting stress than fathers. This is often the case, as in our study, 54 % of mothers in the clinical sample perceived more parenting stress than fathers.

For diagnosis, the value of questionnaires may be limited. Especially, when only one parent is used as informant, parenting stress may be of influence on ratings of externalizing behavior problems in children. Therefore, parenting stress questionnaires should be part of the assessment of externalizing behavior problems in preschool children and information from the different rating scales should be weighed by clinicians in a proper way. For example, the clinician has to investigate whether parents’ ratings may be the result of a stressful perspective, or, in the case of discrepancy, whether this discrepancy reflects real-life differences in behavior of the child or different levels of parenting stress. Clinicians should evaluate and interpret the outcomes in the context of a multi-informant strategy including a clinician-based severity rating.
